# Contractile State Dependent Sarcomere Length Variability in Isolated Guinea-Pig Cardiomyocytes

**DOI:** 10.3389/fphys.2022.857471

**Published:** 2022-04-04

**Authors:** Oleg Lookin, Anastasia Khokhlova, Tatiana Myachina, Xenia Butova, Olivier Cazorla, Pieter de Tombe

**Affiliations:** ^1^ Institute of Immunology and Physiology, Ural Branch of Russian Academy of Sciences, Yekaterinburg, Russia; ^2^ Laboratoire “Physiologie et Médecine Expérimentale du Coeur et des Muscles”, Phymedexp, INSERM—CNRS - Montpellier University, Montpellier, France; ^3^ Department of Physiology and Biophysics, University of Illinois at Chicago, Chicago, IL, United States

**Keywords:** sarcomere length, intracellular variability, transmural heterogeneity, guinea-pig cardiomyocytes, normal distribution

## Abstract

Cardiomyocytes contract keeping their sarcomere length (SL) close to optimal values for force generation. Transmural heterogeneity in SL across the ventricular wall coordinates the contractility of the whole-ventricle. SL heterogeneity (variability) exists not only at the tissue (macroscale) level, but also presents at the level of a single cardiomyocyte (microscale level). However, transmural differences in intracellular SL variability and its possible dependence on the state of contraction (e.g. end-diastole or end-systole) have not been previously reported. In the present study, we studied three aspects of sarcomere-to-sarcomere variability in intact cardiomyocytes isolated from the left ventricle of healthy guinea-pig: 1) transmural differences in SL distribution between subepi- (EPI) and subendocardial (ENDO) cardiomyocytes; 2) the dependence of intracellular variability in SL upon the state of contraction; 3) local differences in SL variability, comparing SL distributions between central and peripheral regions within the cardiomyocyte. To characterize the intracellular variability of SL, we used different normality tests for the assessment of SL distributions, as well as nonparametric coefficients to quantify the variability. We found that individual SL values in the end-systolic state of contraction followed a normal distribution to a lesser extent as compared to the end-diastolic state of contraction (∼1.3-fold and ∼1.6-fold in ENDO and EPI, respectively). The relative and absolute coefficients of sarcomere-to-sarcomere variability in end-systolic SL were significantly greater (∼1.3-fold) as compared to end-diastolic SL. This was independent of both the transmural region across the left ventricle and the intracellular region within the cardiomyocyte. We conclude that the intracellular variability in SL, which exists in normal intact guinea-pig cardiomyocytes, is affected by the contractile state of the myocyte. This phenomenon may play a role in inter-sarcomere communication in the beating heart.

## Introduction

Sarcomeres form the basic contractile unit of cardiac muscle. The coordinated activation of hundreds of sarcomeres that are mechanically and structurally coupled to each other underlies the Frank-Starling law of the heart (de Tombe and ter Keurs, 2016). Cardiomyocytes contract keeping the end-diastolic sarcomere length (SL) and end-systolic SL close to their optimal values at the peak of the length-tension curve (de Tombe et al., 2010). Therefore, the measurement of SL during key states of cardiomyocyte contraction (end-diastole and end-systole) and its detailed analysis are crucial in the study of the mechanical behavior of the heart.

Cardiomyocytes from distinct myocardial layers of the ventricular wall, sub-endocardial (ENDO) and sub-epicardial (EPI), differ both in their SL properties as well as force generation (Natali et al., 2002; Wan et al., 2003; Cazorla et al., 2005; Stones et al., 2007; Ait Mou et al., 2008; Bollensdorff et al., 2011; Khokhlova et al., 2018). Heterogeneity in sarcomere dynamics also exists among cardiomyocytes in a small tissue sample taken from one region of the heart, e.g. EPI layer (Clark and Campbell 2019; Pitoulis et al., 2020; Clark et al., 2021). Recent studies have shown that SL non-uniformity in the ventricles exists not only at the tissue (macroscale) level, but also present at the level of single cardiomyocyte (microscale level) (Sarai et al., 2002; Serizawa et al., 2011; Shintani et al., 2014; Kobirumaki-Shimozawa et al., 2016; de Souza Leite and Rassier 2020; Kobirumaki-Shimozawa et al., 2021). At the microscale level, the variability in SL may regulate force developed by a cardiomyocyte (de Souza Leite and Rassier 2020; Kobirumaki-Shimozawa et al., 2021). Indeed, it was shown that subtle changes in SL (∼100 nm) result in noticeable changes in the developed tension (Fukuda et al., 2001; Serizawa et al., 2011; Kobirumaki-Shimozawa et al., 2014). However, the transmural aspects of intracellular variability and its possible dependence on the state of myofilament activation (state of contraction) have not been previously reported.

In the present study, we compared sarcomere-to-sarcomere variability in the end-diastolic and end-systolic SL in mechanically unloaded isolated guinea-pig cardiomyocytes obtained from ENDO and EPI layers of the left ventricle (LV). To examine whether the variability in SL may differ within a single cardiomyocyte, we also compared individual SL distributions in two distinct regions (central and peripheral) of single cardiomyocytes. We found that individual SL values in the end-systolic state of contraction follows normal distribution to a lesser extent as compared to the end-diastolic state of contraction (∼1.3-fold and ∼1.6-fold in ENDO and EPI, respectively). The relative and absolute coefficients of sarcomere-to-sarcomere variability in end-systolic SL were ∼1.3-fold higher compared to end-diastolic SL. This was independent of both the transmural region across the left ventricle and the intracellular region within the cardiomyocyte. We conclude that the intracellular variability in SL, which exists in normal intact guinea-pig cardiomyocytes, is affected by the contractile state of the myocyte and may play a role in the inter-sarcomere communication in the beating heart.

## Materials and Methods

### Isolation of Cardiomyocytes

Male and female guinea-pigs, weighing 350–500 g, were obtained from the institutional vivarium and maintained under standard conditions (12 h light/dark cycle with ad libitum access to water and food). The animals were injected intramuscularly with heparin sodium (5000 IU/kg, Ellara, Russia) to prevent blood clotting in coronary vessels, anesthetized 30 min later with Zoletil-100 (0.3 ml/kg body weight, Virbac, Carros, France) and 2% Xylazine (1 ml/kg body weight, Alfasan, Netherlands) and rapidly euthanized 15–20 min later by exsanguination following removal of the heart.

The removed heart was immediately flushed with heparin-containing cold (10–15°C) Krebs-Henseleit bicarbonate buffer (see detailed composition in Supplementary Table S1). Next, the heart was quickly cannulated *via* the aorta and perfused using a Langendorff apparatus with a sequence of three solutions equilibrated with 95% O_2_ + 5% CO_2_ at a rate of 5 ml/min at 36°C. The perfusion was started with heparinized Krebs-Henseleit bicarbonate buffer for 5 min after the heart has started normal beating (80–110 beat/min). Next, perfusion was switched to low Ca^2+^ Krebs-Henseleit buffer for 15 min (causing the heart to stop beating). Next, the heart was perfused with enzyme solution, containing 0.2 mg/ml collagenase (∼300 U/mg; CLS-2, Worthington, United States) and 0.06 mg/ml protease XIV (∼3.5 U/mg) for 6 min. After the appearance of viscous drops at the heart apex, the heart was transferred to a Petri dish with enzyme solution. The LV was gently digested by the Langendorff-free injection technique (Butova et al., 2021) at a rate of 6.0–7.0 ml/min and 36°C. After complete digestion, thin ENDO and EPI layers (<1/3 width of the LV free wall) were obtained using fine scissors and forceps. Then ENDO and EPI tissues were exposed to mechanical disruption, re-suspended with Stopping buffer (Krebs-Henseleit bicarbonate buffer with 5 mg/ml BSA and without collagenase or protease), filtered, and gradually adjusted to 1.8 mM extracellular Ca^2+^ concentration. The final suspensions with single cardiomyocytes were stored in oxygenated (100% O_2_) HEPES-buffered Tyrode solution (in mM: 140 NaCl, 5.4 KCl, 1.0 MgSO_4_, 10.0 HEPES, 11.1 d-Glucose, 1.8 CaCl_2_, pH 7.35 with NaOH) at 22–24°C and used within 4–6 h. For measurements of sarcomere dynamics, suspensions of ENDO and EPI cardiomyocytes were diluted in fresh HEPES-buffered Tyrode and transferred to the experimental chamber. Measurements were performed at 30°C and pacing rate of 1 Hz using an electronic pacing device (MyoPacer, IonOptix, Ireland). Unless otherwise noted, all chemicals and reagents were purchased from Sigma-Aldrich (St Louis, MO, United States).

### Optical Measurement of Individual Sarcomere Lengths in a Single Intact Cardiomyocyte

**FIGURE 1:**
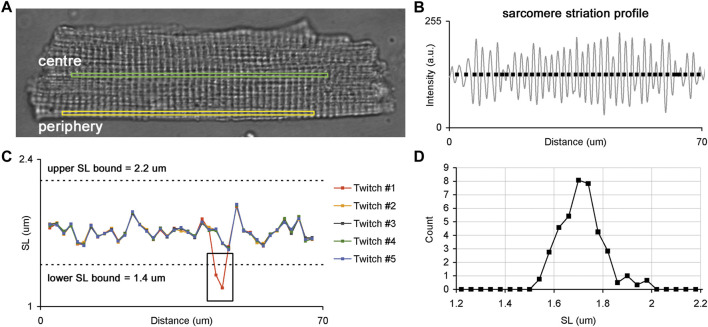
Sarcomere striation profile in a single isolated membrane intact guinea-pig cardiomyocyte. **(A)** Whole-cell image of a single cardiomyocyte with two ROIs selected for the analysis of sarcomere striation profiles in two different regions: central (green) and peripheral (yellow) regions of the same cell. **(B)** Example of the sarcomere striation profile observed in one ROI and one frame. The grey curve follows the sarcomere striation profile, and black rectangles show the positions of individual sarcomeres along the ROI beginning from the left edge. **(C)** Plots of individual SL values (here—end-diastolic) as a function of their position along the ROI. The SL values of each cardiomyocyte were derived from corresponding frames for end-diastole or end-systole, where several consecutive twitches were superimposed to obtain the SL values. The black box demonstrates where an error of SL measurement occurred because of the instability in the sarcomere detection in some frames. Such fragments were excluded from further analysis. Dashed lines show lower and upper SL bounds (1.4 and 2.2 µm for end-diastolic SL values, respectively). **(D)** SL distribution obtained from data displayed in “C”. See text for more details.

A laser confocal scanning microscopy (LSM 710, Carl Zeiss, Germany) was used to measure the sarcomere striation profile in a single isolated cardiomyocyte contracting in mechanically unloaded conditions. The optical settings were as follows: transmitted light mode (T-PMT channel); phase-contrast imaging mode with a 63x oil-immersion objective (Plan-Apochromat 63x/1.40 Oil DIC M27); excitation by 488 nm (2–3% of maximal power). The physical spatial resolution of the measured sarcomere striation profile using the given optical settings was ∼0.12 µm. The acquisition settings for time-series measurements of a sarcomere striation profile in electrically paced cardiomyocytes were set as follows: region-of-interest (ROI) mode, where the horizontal ROI size was adjusted optimally for the selected optical settings (∼1,000 pixels) along the long cell axis (ROI width) and the vertical ROI size was 2-3 pixels along the short cell axis (ROI height, Figure 1A); single frame scan time was 2–4 ms.

To compare individual sarcomere lengths in two different regions of a single cardiomyocyte, we sequentially acquired time-series of sarcomere striation profiles in the central and peripheral ROI oriented along the long cell axis (Figure 1A). The ROI sizes of central and peripheral regions were set to be equal for each cardiomyocyte. Five or more regular twitches of a cardiomyocyte were analyzed to obtain its sarcomere striation profile under steady-state conditions of the cardiomyocyte.

### Offline Processing of Sarcomere Striation Profile to Retrieve Individual Sarcomere Lengths

The time-series of sarcomere striation profiles measured in central and peripheral regions were processed offline only for regularly striated images. First, for each twitch, the corresponding frames were identified during the end-diastolic and end-systolic states of contraction. The raw unfiltered sarcomere striation profile was obtained as the dependence of the pixel intensity on the distance from the left frame edge, where brighter pixels had higher intensities. Next, a high-frequency filter was applied to each raw sarcomere striation profile to find non-periodical oscillation. This non-periodical component was then subtracted from the raw sarcomere striation profile, and the resulting high-frequency periodical oscillations, which are related to sarcomeric striation, were processed (see Supplement for further details). To measure individual SL we determined the positions at which the sarcomere striation profile curve either crossed its baseline (as shown in Figure 1B) or achieved its local maximum or minimum. The intersection points of the sarcomere striation profile curve with the baseline were determined by interpolation between two neighboring positions. The local maximum/minimum positions were calculated from second order polynomial approximation of three neighbor points: the point of local maximum/minimum and the points before and after this point. The intervals between two neighbor positions of individual sarcomeres in the sarcomere striation profile were interpreted as the lengths of individual sarcomeres. The number of individual sarcomeres in a sarcomere striation profile was ≥35 in each ROI at both end-diastolic and end-systolic states.

The end-diastolic and end-systolic SL values of each cardiomyocyte were derived from frames depicting end-diastolic or end-systolic states and several consecutive twitches were superimposed to reveal out-of-range values (see a black rectangle in Figure 1C). These out-of-range values were discarded from further analysis and the remaining SL values were processed as follows: SL values for each position along the ROI were averaged for several twitches and this averaged value was then interpreted as an individual SL value. Additionally, SL values beyond the lower and upper bounds (respectively, 1.4 and 2.2 µm for end-diastolic SL values and 1.2 and 2.2 µm for end-systolic SL values) were removed from an individual SL value set. The obtained set of individual SL values was further used to characterize their variability across the ROI and the parameters of distribution in central and peripheral regions from ENDO and EPI cardiomyocytes.

After the sarcomere striation profile processing, additional criteria were imposed to include a cardiomyocyte into further analysis: 1) an averaged end-diastolic inclusion SL criterion was set to: 1.7 µm ≤ SL ≤ 1.9 µm, and 2) a fractional sarcomere shortening (sarcomere shortening divided by the end-diastolic SL) criterion was set to: 5% ≤ sarcomere shortening ≤ 20%.

### Statistical Analysis

Data analyses were carried out with GraphPad Prism 7.0 (GraphPad Software, CA, United States) and custom-made software EqapAll6. We used one-sample Shapiro-Wilk, and one- and two-sample Anderson-Darling statistical tests to assess the normality of SL value distributions. For all tests, the hypothesis of normality was rejected at a *p*-value of <0.05.

For each SL set we used non-parametric measures: median, median absolute deviation (MAD), and MAD divided by median (MADM). A two-way and three-way ANOVA with Sidak multiple comparison tests were used for statistical analyses between the parameters. The parameters of all analyzed sets were distributed normally (checked using Shapiro-Wilk normality test) and had similar variance between groups (checked using Bartlett’s test). A *p*-value of <0.05 was considered to indicate a significant difference between the parameters.

## Results

### Assessment of Individual Sarcomere Length Distributions

**FIGURE 2:**
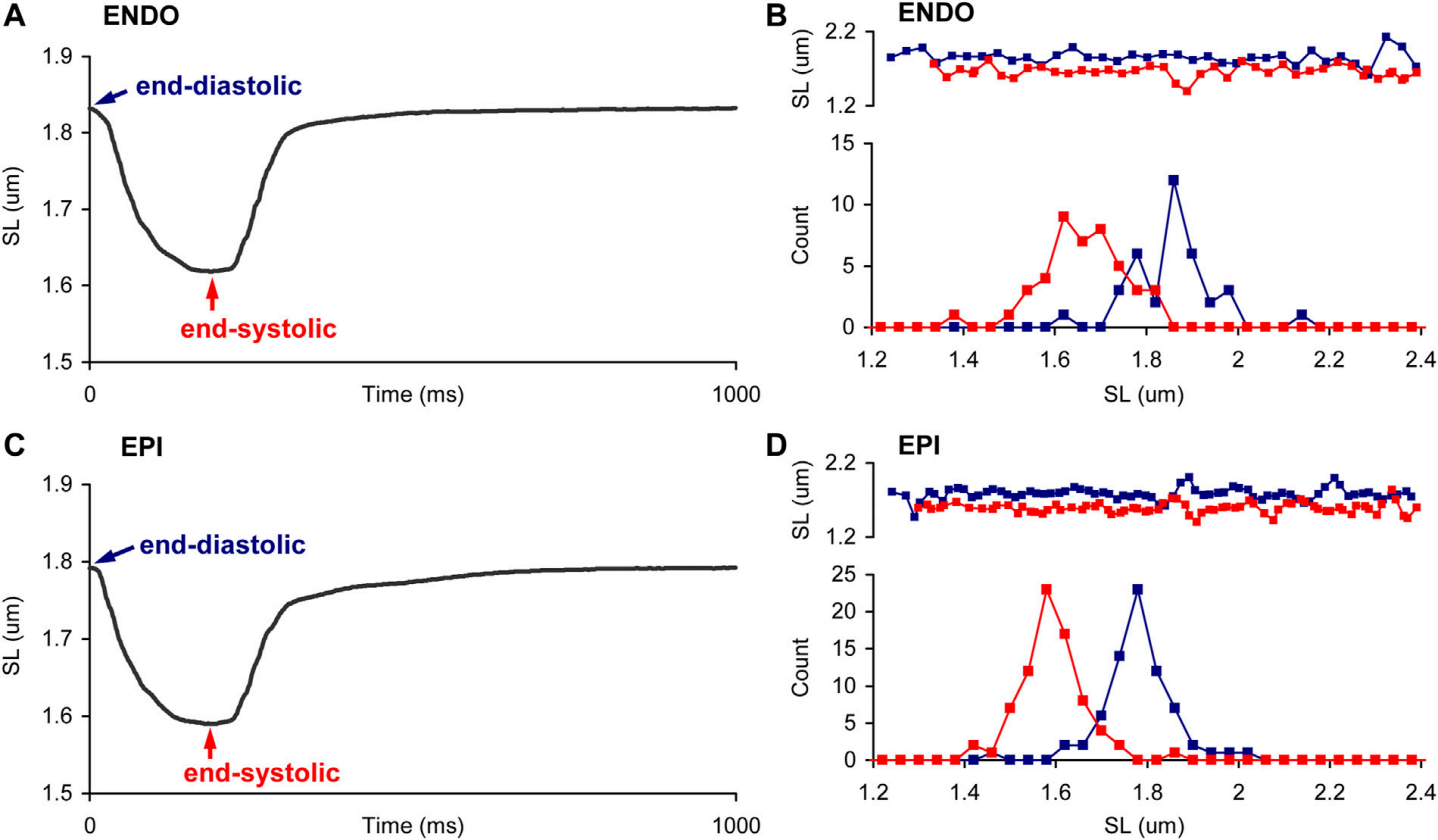
Sarcomere length distribution in ENDO and EPI myocytes during the contraction. **(A,C)** Representative SL changes in time, averaged from one ROI in a single paced ENDO **(A)** and EPI **(C)** cardiomyocyte at 1 Hz. **(B,D)**. Representative distributions, obtained in end-diastole (EDSL, blue) and end-systole (ESSL, red), in ENDO **(B)** and EPI **(D)** cardiomyocytes. Top panels show plots of individual SL values as function of their position along the ROI. The SL values of each cardiomyocyte were derived from frames recorded during end-diastole or end-systole; several consecutive twitches were superimposed. The distributions of individual SL sets are plotted with a bin size of 0.04 µm.

**FIGURE 3:**
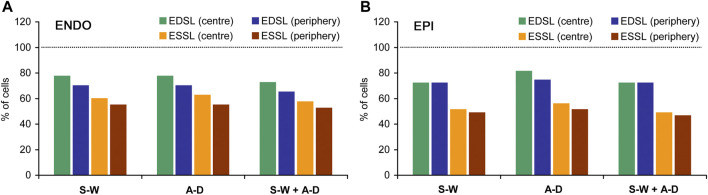
The percentage of ENDO **(A)** and EPI **(B)** cardiomyocytes in which distributions of individual end-diastolic SL (EDSL) and end-systolic (ESSL) passed different normality tests. S-W: Shapiro-Wilk one-sample test, A-D: Anderson-Darling one-sample test; S-W + A-D: Shapiro-Wilk one-sample test together with Anderson-Darling one-sample test (both tests passed). The number of ENDO cardiomyocytes *n* = 40, the number of EPI cardiomyocytes *n* = 43 (from 8 hearts). Dotted horizontal lines correspond to the level where all cells passed a test (100%).

Figure 2 shows representative distribution plots for the end-diastolic and end-systolic SL values of paced single ENDO and EPI cardiomyocytes. This example demonstrates that the shape of individual SL distribution can be substantially affected by the state of contraction and that intercellular variability can exist as well (compare ENDO and EPI myocytes in Figures 2B,D, respectively). Therefore, it was needed to analyze individual SL sets for each myocyte independently of other cells. To fulfill the task, we used different normality tests to assess whether each individual SL set follows the normal distribution (Figure 3). The Shapiro-Wilk and Anderson-Darling tests showed virtually the same inclusion rate, independent of the state of contraction (end-diastolic or end-systolic) or intracellular region (central or peripheral). Regardless of test, the passage rate was consistently lower for individual SL sets measured in a cell at the end-systolic state, compared to the SL set in the same cell at the end-diastolic state (Figure 3).

On average for pooled data (ENDO + EPI, central + peripheral intracellular regions), the passage rates for Shapiro-Wilk and Anderson-Darling one-sample tests were, respectively, 72.9 ± 3.2 and 75.8 ± 4.8% for end-diastolic state of contraction, while the passage rates for end-systolic state were, respectively, 53.8 ± 4.9% and 56.1 ± 4.7%. If both Shapiro-Wilk and Anderson-Darling tests for the given cell were required to satisfy (for SL sets measured either in central or in peripheral intracellular region), the passage rate was 70.4 ± 3.6% and 51.3 ± 4.8% for end-diastolic and end-systolic state of contraction, respectively. According, the percentage of cells in which end-diastolic and end-systolic SL distributions did not pass both Shapiro-Wilk test and Anderson-Darling test was 21.7 ± 4.2% and 41.5 ± 4.8%, respectively (mixed data for ENDO + EPI, central + peripheral intracellular regions). Moreover, if the passage of Shapiro-Wilk and Anderson-Darling one-sample tests in both central and peripheral intracellular regions was required, the passage rate further decreased to 50.4 ± 7.6% and 29.1 ± 4.9% for end-diastolic and end-systolic state of contraction, respectively. The Anderson-Darling two-sample test for two individual SL sets, which were measured in the central and peripheral intracellular regions of the same cell in the same state, showed a higher passage rate: 84.5 ± 4.3% and 80.9 ± 5.9% for end-diastolic and end-systolic state of contraction, respectively. On average, for all four types of normality tests (Shapiro-Wilk one-sample test only, Anderson-Darling one-sample test only, Shapiro-Wilk + Anderson-Darling one-sample tests, and Anderson-Darling two-sample test), the number of cells in which end-systolic SL distributions pass normality tests was ∼1.3-fold lower in ENDO cells and ∼1.6-fold lower in EPI cells compared to end-diastolic SL distributions. The percentage of cells in which individual SL distributions passed Shapiro-Wilk test were close to that for Anderson-Darling test, while this percentage was lower when these tests were applied together.

Thus, our analysis showed that individual SL values in a single cardiomyocyte can be distributed by a function other than normal. Therefore, in our further analysis we used non-parametric measures of absolute and relative sarcomere-to-sarcomere variability in SL (MAD and MADM, respectively).

### Lack of Regional Differences in Characteristics of Individual Sarcomere Length Sets

**FIGURE 4:**
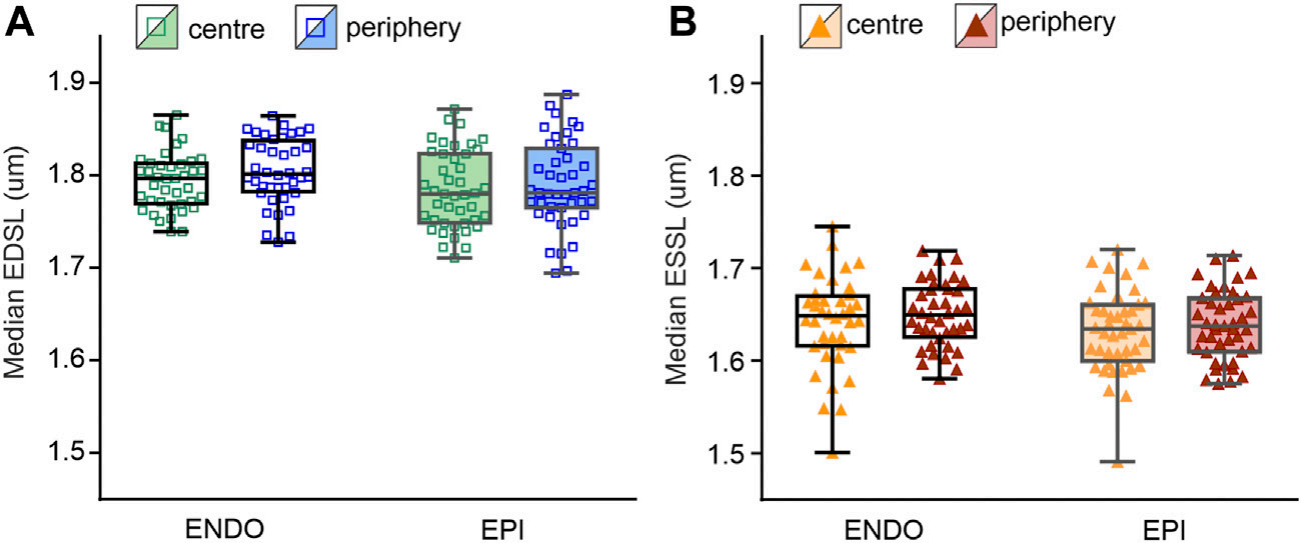
Median SL values obtained from individual SL sets in central and peripheral regions of ENDO (*n* = 40) and EPI (*n* = 43) cardiomyocytes (from 8 hearts). **(A)**: Median end-diastolic SL (EDSL) values. **(B)** Median end-systolic SL (ESSL) values. Two-way ANOVA was used for all comparisons. None of the comparisons revealed any statistically significant differences.

To reveal regional differences in end-diastolic and end-systolic SL between ENDO and EPI cardiomyocytes or between central and peripheral intracellular regions, we compared median SL values obtained from individual SL sets (see Supplementary Figure S2, S3 for sarcomere twitches and distribution plots of end-diastolic and end-systolic SL). We found no significant differences in the median values between ENDO and EPI cardiomyocytes or between central and peripheral regions in each transmural region (Figure 4). End-diastolic SL of ENDO or EPI cardiomyocytes was ∼1.8 µm, while the end-systolic SL was ∼1.64 µm.

To access regional differences in the variability of individual SL, we analyzed the measures of absolute variability (median absolute deviation, MAD) and relative variability (MAD divided by median value, MADM) obtained from individual SL sets. No significant differences in the mean values of MADM or MAD of end-diastolic or end-systolic SL were found between ENDO and EPI cells or between central and peripheral intracellular regions (see Supplementary Figure S4, S5). Note that mean values of relative or absolute variability were not different from each other for each cellular or sub-cellular region.

**FIGURE 5:**
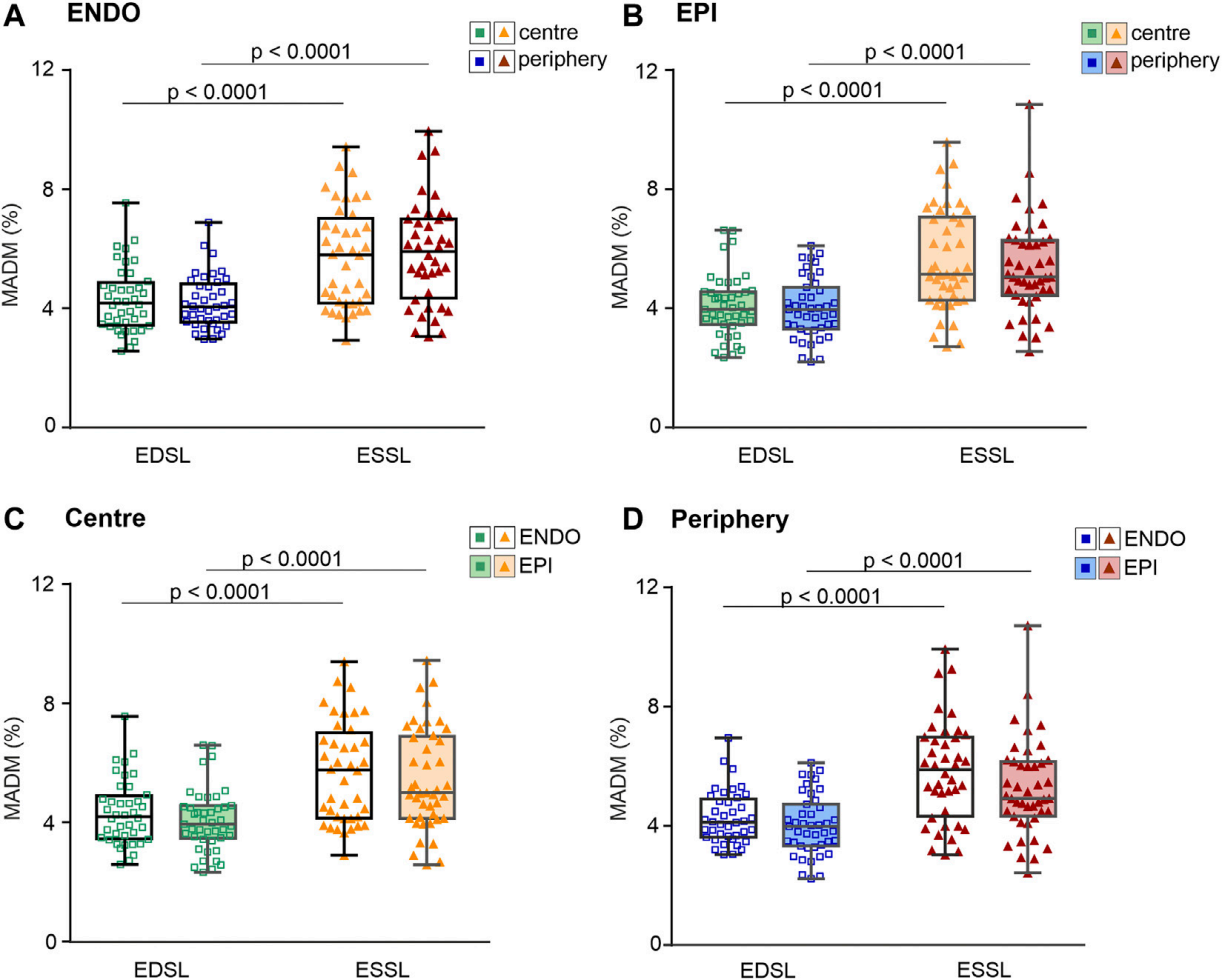
Median absolute deviation divided by median value (MADM, %) obtained for individual SL sets measured at different contraction states (end-diastole, EDSL, or end-systole, ESSL) in central and peripheral intracellular regions of ENDO (*n* = 40) and EPI (*n* = 43) cardiomyocytes (from 8 hearts). **(A)**: MADM for SL distributions in ENDO cardiomyocytes. **(B)**: MADM for SL distributions in EPI cardiomyocytes. **(C)**: MADM for SL distributions in the central intracellular region of ENDO and EPI cardiomyocytes. **(D)**: MADM for SL distributions in the peripheral intracellular region of ENDO and EPI cardiomyocytes. *p* < 0.0001, three-way ANOVA was used for all comparisons. Note that the top and bottom panels contain the same data sets, but displayed in different compositions depending on the comparison factor (top panels = ENDO *vs*. EPI, bottom panels = centre *vs*. periphery).

Finally, we assessed the effect of contraction state on the SL distribution variability. For each cellular or sub-cellular region, we found that relative and absolute coefficients of variability in end-systolic SL were significantly greater compared to coefficients of variability in end-diastolic SL (*p* < 0.001, Figure 5; see also Supplementary Figure S5). Note that top and bottom panels in Figure 5 contain the same data sets exploring different compositions depending on the comparison factor, i.e. top panels for ENDO *vs*. EPI, and bottom panels for centre *vs*. periphery. The significance of this difference was invariant to the intracellular region (central or peripheral) or whether we used pooled data from both regions. For example, for pooled data from central and peripheral intracellular regions the mean value of MADM in ENDO myocytes was 4.28 ± 0.99% and 5.80 ± 1.69% for end-diastolic and end-systolic states of contraction, respectively (*p* < 0.0001). Likewise, for pooled data obtained in EPI myocytes, the mean value of MADM was 4.02 ± 1.02% and 5.40 ± 1.64% for end-diastolic and end-systolic states of contraction, respectively (*p* < 0.0001). Therefore, MADM was ∼1.35-fold greater (CV was ∼1.3-fold greater) for the end-systolic state compared to the end-diastolic state, indicating that sarcomere-to-sarcomere SL variability increases during contraction.

**FIGURE 6:**
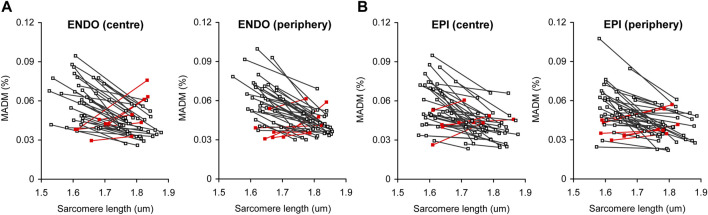
Correlation plots between actual median value of sarcomere length and the extent of sarcomere-to-sarcomere SL variability (evaluated by median absolute deviation divided by median, MADM) for end-diastole (higher SL value for each individual line) and end-systole (lower SL value for each individual line). **(A)** Pool of ENDO cells from central and peripheral intracellular regions (*n* = 40). **(B)** Pool of EPI cells from central and peripheral intracellular regions (*n* = 43). Red lines show cells with inverted relationship between median SL value and MADM value [∼15% of total number of cells for pooled ENDO and EPI cardiomyocytes (from 8 hearts)].

To demonstrate the correlation between the contractile state of a cardiomyocyte and the extent of sarcomere-to-sarcomere SL variability in a particular cardiomyocyte, we plotted end-diastolic (for resting state) or end-systolic (for maximally contracting state) mean SL values against MADM values for the corresponding individual SL values in each individual cell (Figure 6). Most of the cells in ENDO and EPI pools show increased variability in SL at the transition from the end-diastolic to the end-systolic state of the twitch; the correlation was significant at *p* < 0.0001 (correlation analysis by Pearson’s coefficient). A minority of cardiomyocytes (∼15% for pooled ENDO + EPI cells) showed the opposite effect during the contractile transition with, again, no transmural differences in this value. The separated SL sets for ENDO or EPI pools, either of central or peripheral intracellular regions, showed non-significant differences between each other in the slopes of the relationship between SL and MADM.

## Discussion

### Variability in Sarcomere Lengths in Isolated Contracting Cardiomyocytes

In this study, we used mechanically unloaded membrane intact isolated guinea-pig cardiomyocytes from the subepi- and subendocardial layers of the left ventricle and measured individual sarcomere length (SL) in two intracellular regions (central and peripheral) for each cell in its relaxed state (end-diastole) and at peak contraction (end-systole). The sarcomere-to-sarcomere variability in their lengths was evaluated by absolute and relative non-parametric measures, median absolute deviation (MAD) and MAD divided by median value (MADM), respectively. The main finding is that SL variability is higher at end-systole *vs*. end-diastole, independent of either the transmural origin of a cell or intracellular region. The magnitude of SL variability was found to be non-different between pools of subepicardial (EPI) and subendocardial (ENDO) cardiomyocytes or between two intracellular regions within the same ENDO/EPI cardiomyocyte.

The novelty of this study is that for the first time the variability of individual SL was evaluated in two principally different contractile states of a cardiomyocyte: end-diastole and end-systole. Similar aims have previously been addressed and experimentally fulfilled by use of isolated skeletal and cardiac myofibrils and whole-muscle skeletal preparations (Telley et al., 2006a; Telley et al., 2006b; Pavlov et al., 2009; Moo et al., 2017; Moo and Herzog, 2018), but to our knowledge no such approach was utilized for intact living cardiomyocytes. In a recent study on cardiomyocytes, individual sarcomere lengths were measured in these two states in the cardiomyocytes of the outer surface of the intact heart *in vivo* (Kobirumaki-Shimozawa et al., 2016); however, no characterization of the variability in the measured SL sets has been attempted.

In the present study, we used a confocal scanning system with 63X oil-immersion objective and phase-contrast mode to obtain “bright-field” images of sarcomeres. While conventional bright-field microscopy may not fully satisfy the task of precise sarcomere measurements (Telley et al., 2006a; Kobirumaki-Shimozawa et al., 2016), it has been validated that the sarcomere striation patterns, if they are measured with a simultaneous use of two-photon fluorescence and transmitted light mode by the same scanning microscopy system, highly correlate to each other (Nance et al., 2015). The use of high magnification oil- or water-immersion objectives also allows for better spatial resolution of sarcomeric striation patterns. In addition, limitations of an optical system introduce uniform error in the striation profile and should not substantially affect the discrepancies between SL dispersions obtained under different states (e.g. end-diastole and end-systole). Highly precise evaluation of SL variability can be achieved using Z-disk specific fluorescent labeling, which has been implemented in skinned and cultured cardiomyocytes (Serizawa et al., 2011; Shintani et al., 2014; Kolb et al., 2016) and recently in living mice *in vivo* (Kobirumaki-Shimozawa et al., 2016; Kobirumaki-Shimozawa et al., 2018; Kobirumaki-Shimozawa et al., 2021). It should be noted that, the SL range obtained in our study was found to be similar to that obtained by Z-disk labeling and reported in (Kobirumaki-Shimozawa et al., 2016).

### Functional Role of Sarcomere Length Heterogeneity

Sarcomere length is one of the prime factors regulating the contractile response *via* the mechanism of myofilament length-dependent activation that underlies the Frank-Starling Mechanism (de Tombe and ter Keurs, 2016; Rassier 2017). Nanometer-scale changes in the length of a single sarcomere result in a large change in the contractile response (Fukuda et al., 2001; Serizawa et al., 2011; Kobirumaki-Shimozawa et al., 2014; Rassier 2017). At present, the intracellular inhomogeneity in SL has been shown for isolated skinned and intact cardiomyocytes as well as in the *in-vivo* mouse heart (Sarai et al., 2002; Herzog et al., 2010; Serizawa et al., 2011; Shintani et al., 2014; Kobirumaki-Shimozawa et al., 2016; Shimozawa et al., 2016; Kobirumaki-Shimozawa et al., 2021). This inhomogeneity increases upon the activation of the skeletal and cardiac myofibril (Telley et al., 2006a; Shimamoto et al., 2009; Moo et al., 2017; Rassier, 2017; Moo and Herzog, 2018) or upon an increase in intracellular Ca^2+^ content (Sarai et al., 2002). According to the idea that the adjacent sarcomeres play a “tug of war” during activation of a myofibril (Shimozawa et al., 2016), the systolic stiffening and pre-systolic heterogeneity of sarcomeres (i.e. co-existence of “compliant” and “stiff” sarcomeres) can greatly affect their mechanical communication leading to dynamic changes in SL variability during the contraction-relaxation process (Shimamoto et al., 2009; Azeloglu and Costa, 2010; de Souza Leite and Rassier, 2020; Kobirumaki-Shimozawa et al., 2021). The mechanical communication between in-series sarcomeres is also affected by the passive properties of myofibrils and intracellular matrix (de Souza Leite et al., 2017). On the other hand, SL heterogeneity presents at the level of a single myofibril, whole isolated muscle and *in situ* in living animals (Moo et al., 2017; Moo and Herzog, 2018; Moo and Herzog, 2020), i.e. under highly different mechanical conditions. This supports the notion that intracellular SL dispersion has a specific physiological role rather than that it simply follows the pattern of passive stress/strain. Moreover, the intracellular mechanisms of SL variability may differ between rest and active states of a myocyte (Moo and Herzog, 2020).

It remains poorly studied whether the dispersion of individual SL is linked somehow to the contractile activation of a myocyte and what physiological role this dispersion may represent. In intact skeletal muscle, it has been shown that the *in situ* activation of the myofibrils is accompanied by elevation of SL dispersion (Pavlov et al., 2009; Moo and Herzog, 2018; Moo and Herzog, 2020) which then, however, either remains constant or only slightly decreases during a subsequent period of steady-state activation (Pavlov et al., 2009; Moo and Herzog, 2020). The SL dispersion is higher in a stretched myofibril, but the activation-induced elevation in this dispersion seems to be stretch-independent (Pavlov et al., 2009). The extent of SL dispersion in an activated myofibril may relate to the intrinsic properties of individual sarcomeres, e.g. to their pre-activation level of “off” or super-relaxed myosin motors (McNamara et al., 2015; Piazzesi et al., 2018; Brunello et al., 2020). In turn, dynamic changes in SL dispersion during active shortening may control the relaxation of the whole cell. If true, this in some extent resembles the experimentally validated effect of intercellular heterogeneity on the relaxation properties observed in multicellular tissue samples (Clark et al., 2021).

In cardiac muscle, the activation of myofilaments is more SL-dependent as compared to skeletal muscle (Dobesh et al., 2002; de Tombe et al., 2010). It, therefore, has been proposed that the inter-sarcomeric variability of mechanical properties plays an important role in the regulation of contractile responses of the healthy and, very likely, the diseased heart (Rassier 2017; Kobirumaki-Shimozawa et al., 2021). There are findings that muscle and cardiac dysfunction may be accompanied by alterations in intracellular SL heterogeneity (de Souza Leite et al., 2017; Kagemoto et al., 2018; Timmermann et al., 2019). Importantly, there is also evidence that the variability in individual SL in cardiomyocytes may relate to the local calcium transients in adjacent sarcomeres (Tsukamoto et al., 2016). The increased heterogeneity of SL in the end-systolic state of a cardiomyocyte may be associated not only with local dissimilarities in Ca^2+^ activation of individual sarcomeres but also due to the “steeper” character of cooperative activation of myofilaments in the myocardium: an already “activated” sarcomere shortens more strongly and therefore stretches adjacent non-activated sarcomeres to a higher extent than can occur in the skeletal myocyte. This hypothesis could potentially be tested by evaluation of SL dispersions under different levels of cooperative activation.

Future perspectives in the elucidation of this aspect in cardiac muscle can include studying the dynamic changes in dispersion of not only SL, but also certain sarcomere-related characteristics (maximal rate of shortening, time-to-peak shortening, etc) during a complete contraction-relaxation cycle. The time-course of individual sarcomere(s) can be analyzed to determine to what extent they are distinct from each other and whether a single sarcomere is “synchronized” with the others during the contraction cycle (Kobirumaki-Shimozawa et al., 2021). The time-course analysis of individual sarcomere’s behavior may help to discover a physiological role of SL heterogeneity in the contraction and relaxation processes in healthy and diseased muscle (de Souza Leite et al., 2017). Currently, we can conclude that intracellular SL heterogeneity belongs to the lowest hierarchical level of the phenomenon of myocardial heterogeneity (Cazorla and Lacampagne, 2011; Solovyova et al., 2016), that is, at a smaller level than inter-cellular or the regional tissue level (Clark and Campbell, 2019; Clark et al., 2019; Pitoulis et al., 2020; Clark et al., 2021).

### Lack of Transmural Differences in the Sarcomeric Contractility

In the present study, we did not find significant difference in the median end-diastolic SL or median end-systolic SL between mechanically unloaded guinea-pig cardiomyocytes from ENDO and EPI layers of the left ventricle (see Figure 4 in this manuscript and Supplementary Figure S3). The averaged SL at end-systole was ∼9% smaller than at end-diastole. The amount of SL shortening is slightly lower compared to the data obtained in mouse cardiomyocytes *in vivo* under a natural beat frequency (Kobirumaki-Shimozawa et al., 2016), but is of the same magnitude or even higher compared to the averaged fractional shortening of isolated rat and guinea-pig LV cardiomyocytes, which were mechanically unloaded and paced at a relatively low rate (Natali et al., 2002; Wan et al., 2003; Carneiro-Júnior et al., 2013; Chung and Campbell 2013; Pan et al., 2018).

Likewise, we failed to find transmural differences in the extent of SL variability. It has been shown recently that the subtle inter-cellular (inter-regional) differences in the mechanical properties of living cardiomyocytes can only be revealed employing relatively large sample sizes, containing at least several hundreds of cardiomyocytes (Nollet et al., 2020). For example, based on the examples provided in (Nollet et al., 2020), ∼150 cells are required to reveal inter-regional differences in fractional cell shortening and >500 cells are required to uncover differences in the maximal rate of shortening. Hence, in our study, the number of cells studied might have been be too low to detect inter-regional (ENDO *vs*. EPI) differences in the variability of individual sarcomere lengths.

## Conclusion

The phenomenon of myocardial heterogeneity is manifested not only at the tissue or inter-cellular level, but also at the intracellular level. The extent of intracellular heterogeneity in sarcomere length correlated with the contractile state of the cardiomyocyte and was higher at peak systole compared to diastole. On the other hand, this phenomenon displayed no regional-specific differences, indicating a universal character (independent of cell origin) of SL heterogeneity. Sarcomere-to-sarcomere SL variability and its “sensitivity” to the activation state of a cardiomyocyte may be an important feature, allowing for optimization of the contractile response of the cardiomyocyte to naturally occurring transmural stress and strain gradients in the whole heart. However, elucidation of the functional role of intracellular variability in sarcomere length and other mechanisms of electro-mechanical coupling requires further studies focused on this issue.

## Data Availability

The raw data supporting the conclusions of this article will be made available by the authors, without undue reservation.
