# Supplementary material for: Contractile State Dependent Sarcomere Length Variability in Isolated Guinea-Pig Cardiomyocytes
Source: Front Physiol. 2022 Apr 4;13:857471. doi: 10.3389/fphys.2022.857471 (PMC9013801; doi:10.3389/fphys.2022.857471)
Supplement: Supplementary file 1 [file DataSheet1.DOC]

**Composition of saline solutions used in the Langendorff-perfused whole heart to obtain isolated cardiomyocytes**

Table S1 contains detailed composition of the saline solutions used in the Langendorff-perfused whole heart preparations to obtain isolated cardiomyocytes. The perfusions were done at 36^O^C with continuous saturation by 95% O_2_ + 5% CO_2_.

**Table S1.** The composition of solutions used in the Langendorff-perfused heart during cardiomyocyte isolation procedure.

| Compound | Krebs-Henseleit bicarbonate buffer | Low Ca^2+^  Krebs-Henseleit buffer | Enzyme solution | Stopping buffer |
| --- | --- | --- | --- | --- |
| NaCl (mM) | 121 | 123 | 123 | 123 |
| KCl (mM) | 4.7 | 4.7 | 4.7 | 4.7 |
| MgSO_4_ (mM) | 1.2 | 5 | 5 | 5 |
| KH_2_PO_4_ (mM) | 1.2 | 1.2 | 1.2 | 1.2 |
| NaHCO_3_ (mM) | 19.0 | 10.0 | 10.0 | 10.0 |
| HEPES, free acid (mM) | 10.0 | 10.0 | 10.0 | 10.0 |
| Taurine (mM) | 20.0 | 20.0 | 20.0 | 20.0 |
| Adenosine (mM) | 2.0 | 2.0 | 2.0 | 2.0 |
| d-Glucose (mM) | 11.1 | 11.1 | 11.1 | 11.1 |
| CaCl_2_ (mM) | 1.0 | 0.05 | 0.025 | 0.025 |
| EGTA (mM) | ― | 0.15 | ― | ― |
| Collagenase (mg/mL) | ― | ― | 0.2 | ― |
| Protease XIV (mg/mL) | ― | ― | 0.06 | ― |
| Bovine serum albumin (mg/mL) | ― | ― | ― | 5 |
| pH with NaOH at 35°C, 95% O_2_+5% CO_2_ for 40 min before pH adjustment | 7.25 | 7.0 | 7.25 | 7.25 |

Note: All chemicals purchased from Sigma-Aldrich (USA) except collagenase (CLS-2, Worthingthon, USA).

**Offline processing of sarcomere striation profile to retrieve individual sarcomeres**

The time-series of sarcomere striation profiles, measured in an isolated paced cardiomyocyte, was processed offline by custom-made software EqapAll6 (Figure S1). The processing of time-series of sarcomere striation profile was as follows. First, for each separate twitch, corresponding frames were identified for both end-diastole and end-systole. The raw unfiltered sarcomere striation profiles in the end-diastolic/end-systolic frames were obtained as the pixel intensity drawn against the distance from the left edge of the frame, where darker/brighter pixel has a lower/higher intensity. Next, a high-frequency filter was applied to each raw sarcomere striation profile to find non-periodical oscillation. This non-periodical component was then subtracted from the raw sarcomere striation profile, leaving high-frequency periodical oscillations, which were related to sarcomeric striation (see Figure S1, chart in the middle of the window). To reduce the effect of high-frequency noise in the sarcomere striation profile, the low-frequency filtered sarcomere striation profile was transformed into a cumulative sum of sarcomere striation profiles, where each point was based on the sum of all previous points. This approach substantially reduced the effect of high-frequency irregular fluctuations in the sarcomere striation profile intensity and allowed for better determination of the individual sarcomeres. The filtered and transformed sarcomere striation profile was further processed to find the positions at which the profile curve either crossed its baseline (Figure S1, middle chart), or achieved its local maximum or minimum. In the first case, the “exact” position of baseline crossing was determined by the interpolation between two neighbor actual positions. In the second case, the “exact” position of local maximum/minimum was calculated from a 2^nd^ order polynomial approximation of three neighboring points: the point of local maximum/minimum and the points before and after this point. These calculated values were assumed to be the “exact” positions of individual sarcomeres in the sarcomere striation profile; accordingly, the intervals between two neighboring positions were interpreted as the lengths of individual sarcomeres and used in further analysis. This procedure was independently taken for sarcomere striation profiles obtained from each contractile state-matched frame in several consecutive twitches of a cell and resulted in a corresponding number of sets of individual sarcomere lengths, all plotted as a function of their position in the corresponding sarcomere striation profile (see Figure 1C in the Manuscript).

| 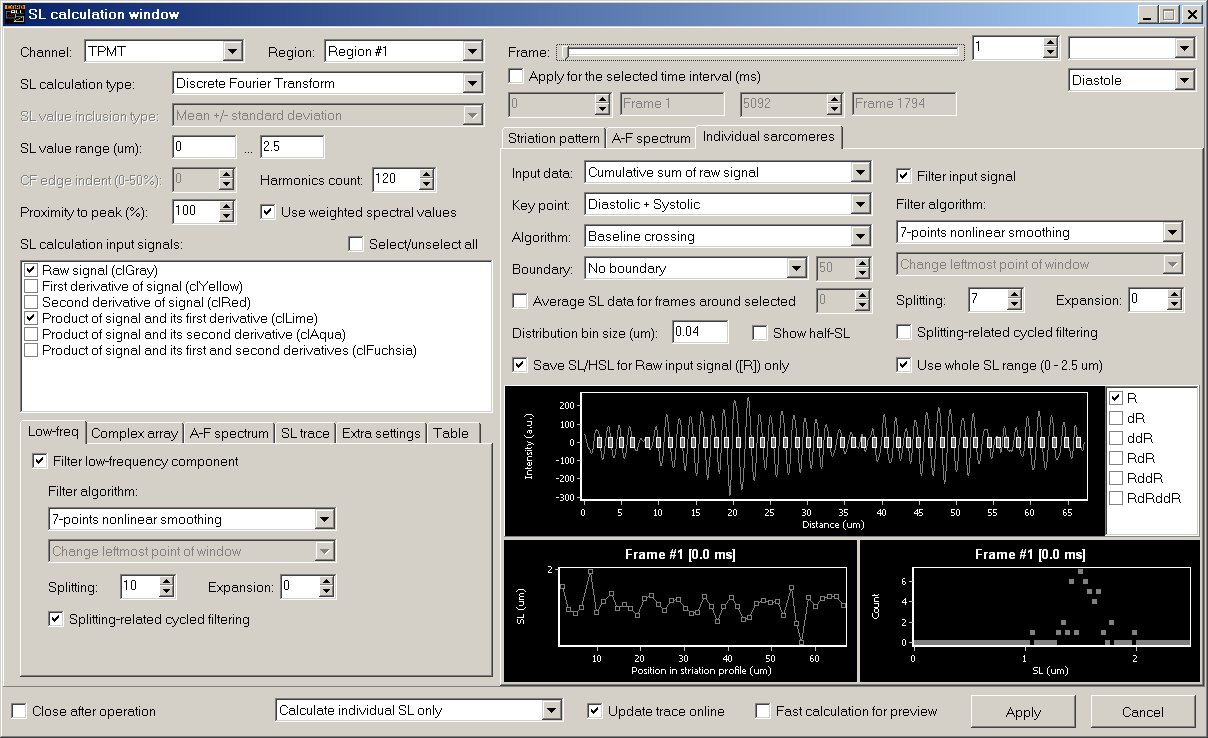 |
| --- |
| **Figure S1.** Screenshot of the software tool used for off-line processing of sarcomere striation profiles. The tool allows for adjustment of multiple settings to obtain individual SL values in a sarcomere striation profile as best as possible. The chart in the middle of window shows the sarcomere striation profile, while bottom charts show individual SL values drawn as function of their distance from the left edge of a region (left chart) and corresponding SL distribution (right chart). The tool was written and used within custom-made software EqapAll6. |

Further, the sets were superimposed to reveal whether in some sets there are out-of-range or improper individual SL. If a measurement was nearly ideal, the sets were highly closer together, otherwise in some sets there were improper positions of SL values (see black rectangle in Figure 1C in the Manuscript). These improper SL values were discarded from further analysis. The remaining SL values were processed as follows: SL values around each single common position were averaged and this averaged value was then interpreted as an individual SL value. Additionally, SL values beyond the lower and upper bounds (in this study, it was 1.4 and 2.2 µm for end-diastolic SL values and 1.2 and 2.2 µm for end-systolic SL values, respectively) were removed from individual SL value set. In the end of this procedure, we obtained a set of individual SL values, which was then used to characterize their variability and the parameters of distribution.

**Lack of transmural differences in characteristics of individual sarcomere length sets**

The individual traces of mean sarcomere shortening, which were obtained for each cell individually in its central or peripheral intracellular region, are shown in Figure S2. The cells shown are those which passed both of two selection criteria: the end-diastolic SL in the range of 1.7-1.9 µm, the fractional shortening in the range of 5-20% of end-diastolic SL.

| 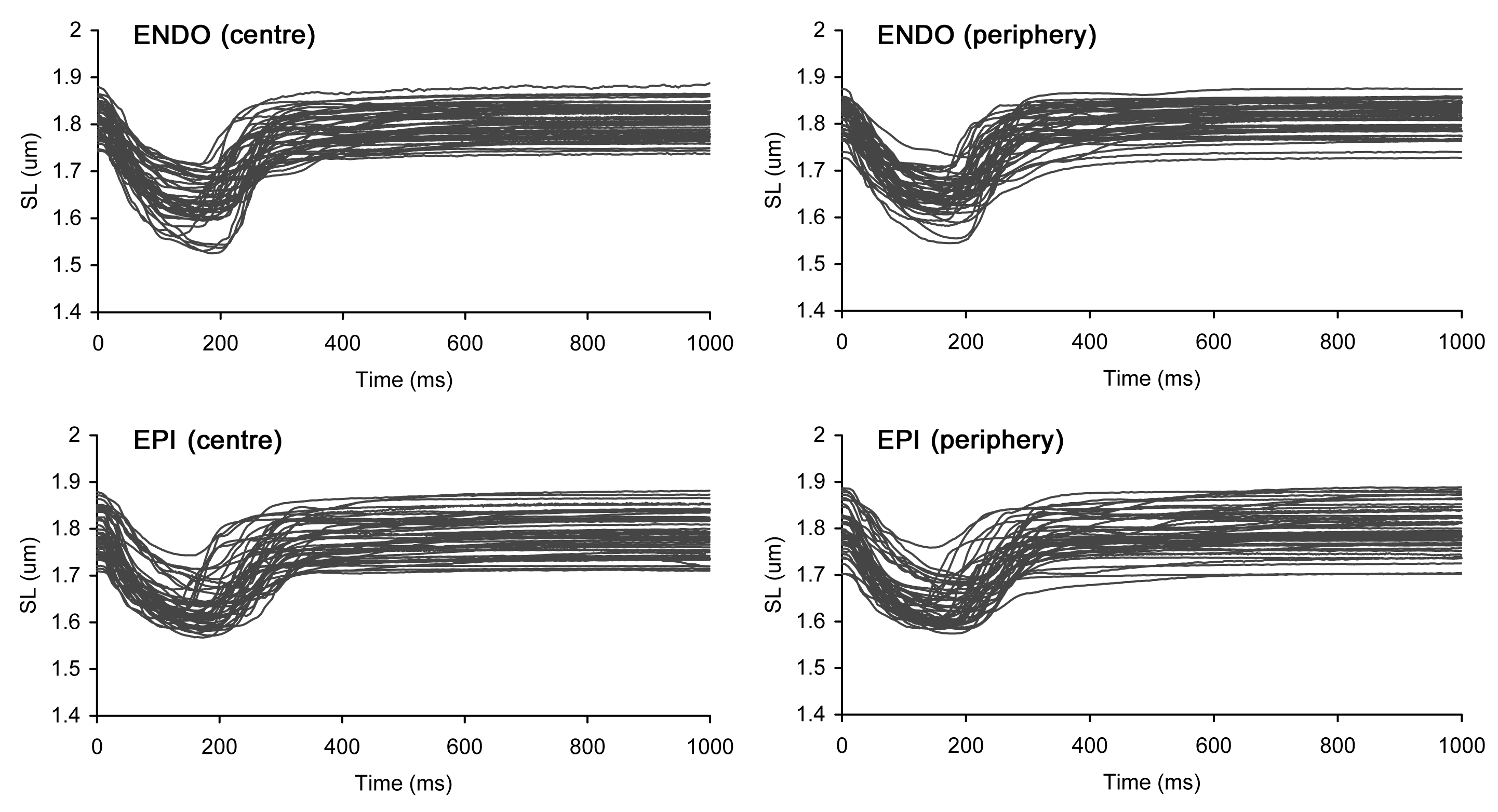 |
| --- |
| **Figure S2.** Superposition of mean sarcomere shortening traces obtained for each isolated cell individually in its central or peripheral intra-cellular regions. The ENDO (*n* = 40) and EPI (*n* = 43) cells were paced at 1 Hz in normal Tyrode at 30°C. |

Figure S3 shows the averaged sarcomere twitches and the averaged distribution plots of end-diastolic and end-systolic SL. We found no significant differences in the median (mean) values between ENDO and EPI cardiomyocytes or between central and peripheral intra-cellular regions for each transmural region.

| 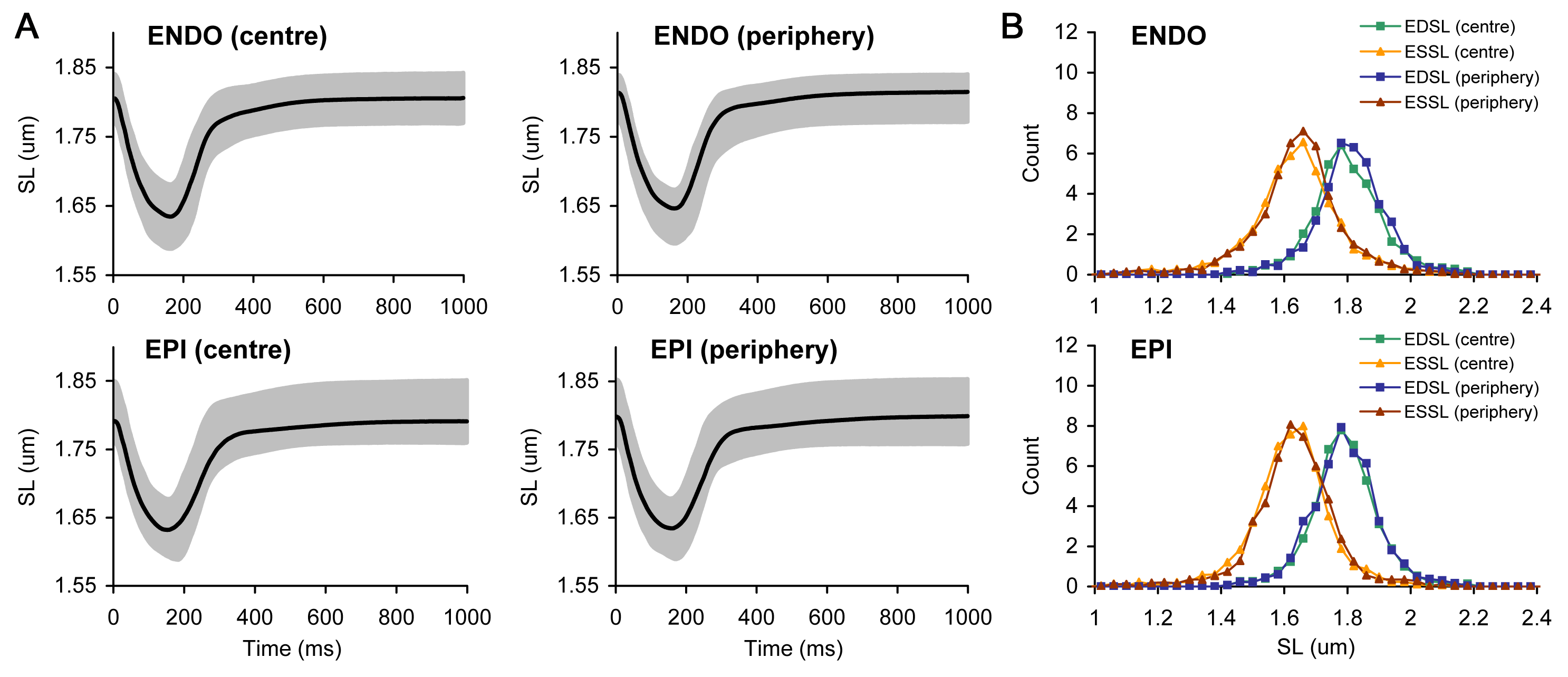 |
| --- |
| **Figure S3.** (A) Average SL changes in central and peripheral regions of ENDO (top panels, *n* = 40) and EPI (bottom panels, *n* = 43) cardiomyocytes (from 8 hearts) paced at 1Hz. The black curve follows the mean SL trace obtained for the corresponding pool of cells; the grey shadow corresponds to the standard deviation of the mean at the time-matched points. (B) Averaged end-diastolic and end-systolic SL distributions (EDSL and ESSL, respectively) obtained in the central and peripheral regions of ENDO and EPI cardiomyocytes. The distributions in panel B are plotted with bin size of 0.04 µm. |

Figure S4 shows that median absolute deviation (MAD) of individual SL sets was not different between ENDO and EPI or between central and peripheral intracellular regions for the same state of contraction (i.e. either end-diastolic or end-systolic).

| 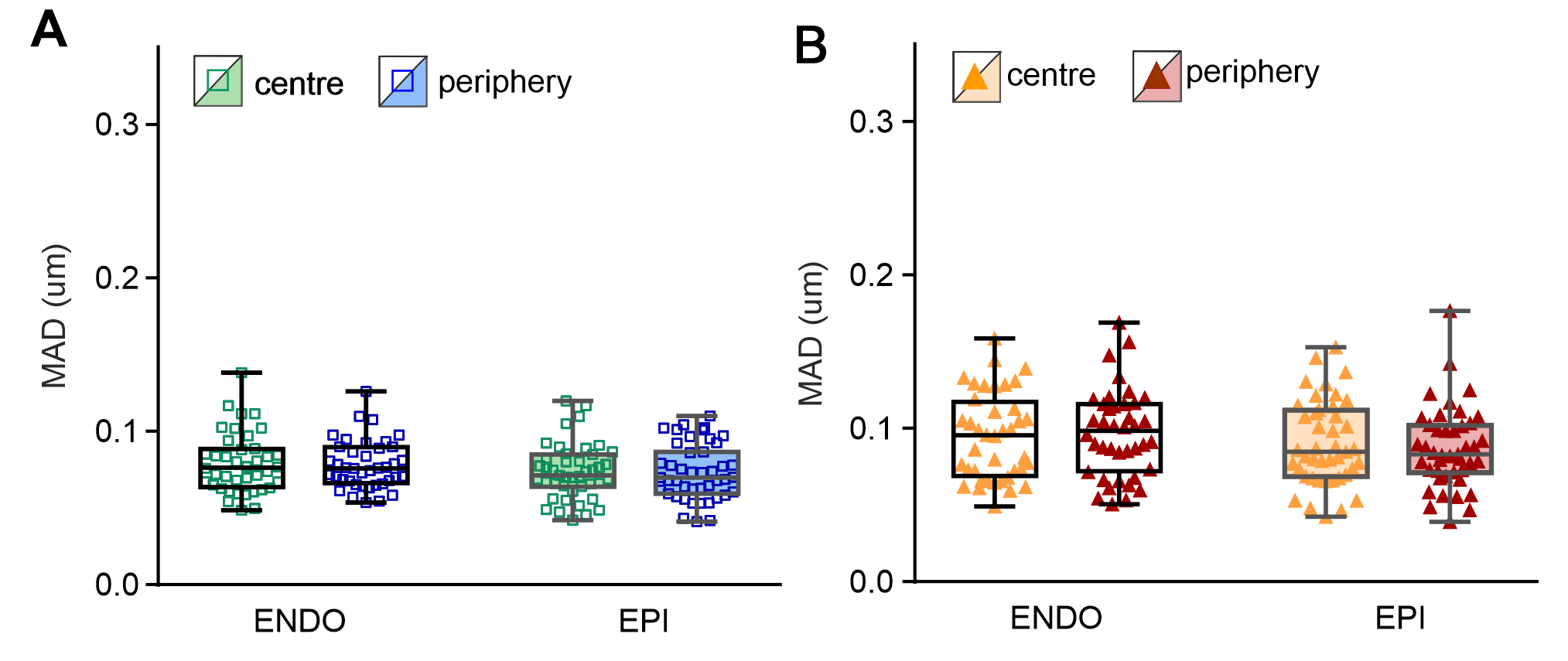 |
| --- |
| **Figure S4.** Median absolute deviation (MAD) obtained from individual SL sets in central and peripheral regions of ENDO (*n* = 40) and EPI (*n* = 43) cardiomyocytes (from 8 hearts). (A): MAD for end-diastolic SL distributions. (B): MAD for end-systolic SL distributions. Two-way ANOVA for all comparisons used. None of the comparisons revealed any statistically significant differences. |

Figure S5 represents the same data but in a different composition, to display that the absolute variability (i.e. evaluated by MAD or SD) in the individual SL revealed significant differences between end-diastolic and end-systolic states of contraction.

| 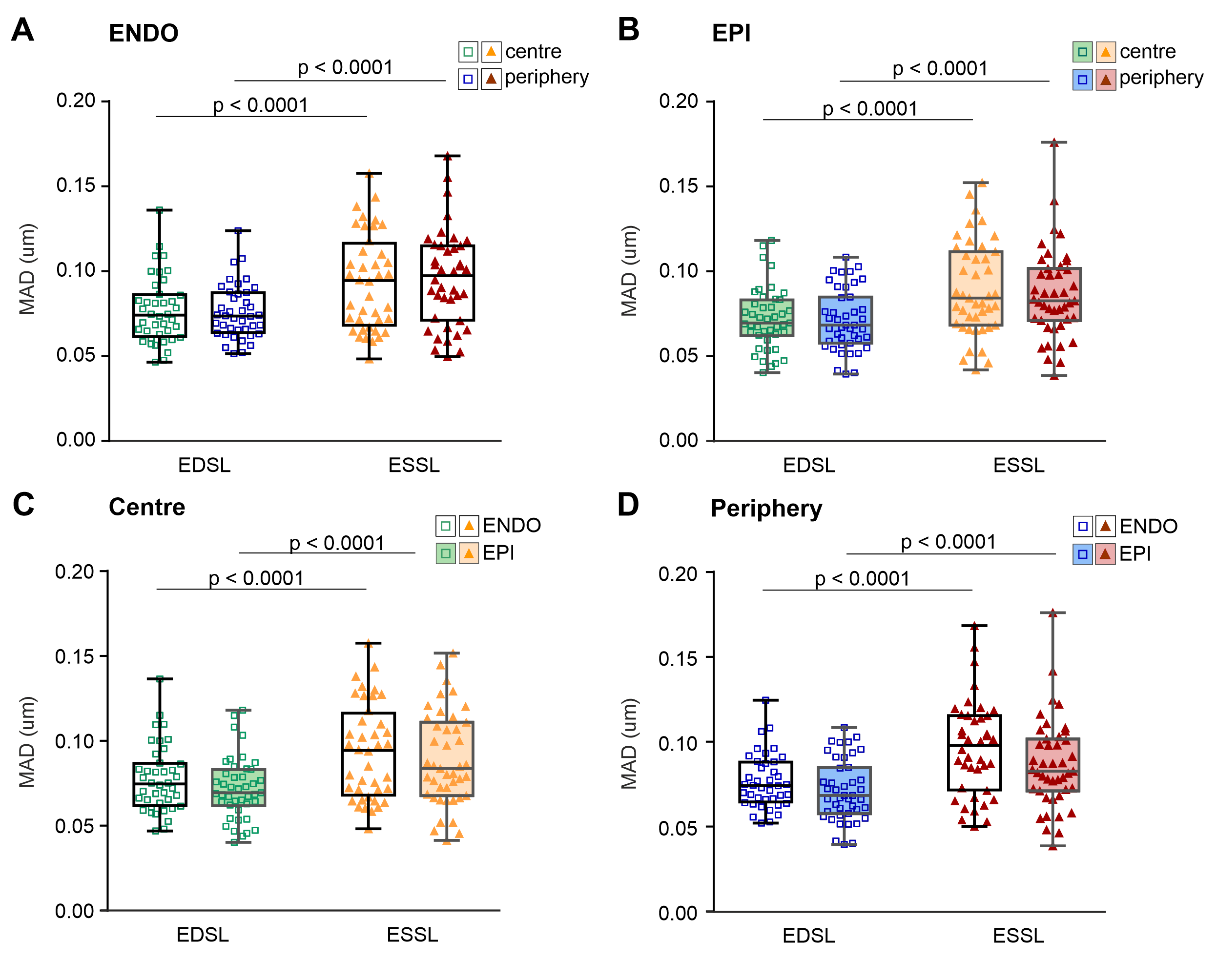 |
| --- |
| **Figure S5.** Median absolute deviation (MAD) obtained from individual SL sets at different contraction states (end-diastolic, EDSL, or end-systolic, ESSL) in central and peripheral regions of ENDO (*n* = 40) and EPI (*n* = 43) cardiomyocytes (from 8 hearts). (A): MAD for SL distributions in ENDO cardiomyocytes. (B): MAD for SL distributions in EPI cardiomyocytes. (C): MAD for SL distributions in the central intracellular region. (D): MAD for SL distributions in the peripheral intracellular region. p < 0.0001, three-way ANOVA for all comparisons used. Note that top and bottom panels contain the same data sets while in different compositions depending on the comparison factor (top panels = ENDO vs EPI, bottom panels = centre vs periphery). |
